# Evolution and genetic characterization of Seoul virus in wild rats *Rattus norvegicus* from an urban park in Lyon, France 2020–2022

**DOI:** 10.1371/journal.pntd.0012142

**Published:** 2024-05-13

**Authors:** Hussein Alburkat, Teemu Smura, Marie Bouilloud, Julien Pradel, Gwendoline Anfray, Karine Berthier, Lara Dutra, Anne Loiseau, Thanakorn Niamsap, Viktor Olander, Diana Sepulveda, Vinaya Venkat, Nathalie Charbonnel, Guillaume Castel, Tarja Sironen

**Affiliations:** 1 Department of Virology, Faculty of Medicine, University of Helsinki, Helsinki, Finland; 2 Department of Veterinary Biosciences, University of Helsinki, Helsinki, Finland; 3 CBGP, IRD, INRAE, CIRAD, Institut Agro, Univ Montpellier, Montpellier, France; 4 CBGP, INRAE, CIRAD, Institut Agro, IRD, Univ Montpellier, Montpellier, France; 5 Department of Veterinary of the zoological garden, Lyon, France; 6 Department of Health of Lyon city, Lyon, France; Colorado State University College of Veterinary Medicine and Biomedical Sciences, UNITED STATES

## Abstract

**Background:**

Seoul virus (SEOV) is an orthohantavirus primarily carried by rats. In humans, it may cause hemorrhagic fever with renal syndrome (HFRS). Its incidence is likely underestimated and given the expansion of urban areas, a better knowledge of SEOV circulation in rat populations is called for. Beyond the need to improve human case detection, we need to deepen our comprehension of the ecological, epidemiological, and evolutionary processes involved in the transmission of SEOV.

**Methodology / Principal findings:**

We performed a comprehensive serological and molecular characterization of SEOV in *Rattus norvegicus* in a popular urban park within a large city (Lyon, France) to provide essential information to design surveillance strategies regarding SEOV. We sampled rats within the urban park of ‘La Tête d’Or’ in Lyon city from 2020 to 2022. We combined rat population genetics, immunofluorescence assays, SEOV high-throughput sequencing (S, M, and L segments), and phylogenetic analyses. We found low structuring of wild rat populations within Lyon city. Only one sampling site within the park (building created in 2021) showed high genetic differentiation and deserves further attention. We confirmed the circulation of SEOV in rats from the park with high seroprevalence (17.2%) and high genetic similarity with the strain previously described in 2011 in Lyon city.

**Conclusion/Significance:**

This study confirms the continuous circulation of SEOV in a popular urban park where the risk for SEOV transmission to humans is present. Implementing a surveillance of this virus could provide an efficient early warning system and help prepare risk-based interventions. As we reveal high gene flow between rat populations from the park and the rest of the city, we advocate for SEOV surveillance to be conducted at the scale of the entire city.

## Author summary

Seoul virus is a zoonotic pathogen primarily carried by rats, and it can cause a serious illness called hemorrhagic fever with renal syndrome in humans. Research into the zoonotic risk associated with this virus is limited although human cases are detected worldwide. We investigated the circulation of SEOV and its genetic evolution in rats from a French urban park, where the virus was detected first in 2010–2012 and a strain characterized in 2011. We have studied 87 wild brown rats distributed non homogeneously all over the park and found that 17.2% of those animals had orthohantavirus-reactive antibodies. Furthermore, the molecular analyses of the virus showed amino acid exchanges over the past decade in this area. This study highlights the potential zoonotic risk posed by SEOV in a popular urban park, where humans are likely to come into contact with wild rats or their excretions. To mitigate this zoonotic risk, targeted surveillance of this virus in rat populations would be beneficial. Additionally, prevention strategies should be designed and communicated with all personnel working in the park.

## Introduction

Seoul virus (SEOV) is an orthohantavirus (*Hantaviridae* family) that is primarily carried by rats, particularly the brown rat (*Rattus norvegicus*) and the black rat (*Rattus rattus*). It is transmitted to humans through contact with infected rats or their droppings, urine, or saliva. In its rodent hosts, SEOV infection is considered persistent and asymptomatic, but in humans it can cause hemorrhagic fever with renal syndrome (HFRS). The severity of the disease can range from mild to severe (1–2% mortality) [1–3].

Because of the widespread distribution of rats, SEOV is found worldwide [4]. It has been detected in Asia, Europe, Africa and the Americas, both in rural and urban regions, although most studies have been conducted in rural regions [4]. Despite its potentially high pathogenicity, the incidence of SEOV infections remains probably underestimated [5]. Several factors are thought to influence the risk of SEOV infection in humans, including urbanization and climate change. A recent study showed that urban greening is another potential factor associated with increasing rat-borne zoonotic diseases in green urban areas [6]. These are critical processes as they have significant impacts on the distribution and expansion of rats worldwide, and on the probability of close contact between rats and humans [7]. Urbanization is a global trend, with more and more people moving into cities each year. As a result, the increase in the density and diversity of urban ecosystems has created favorable conditions for rodents like rats to thrive and settle, potentially leading to a higher incidence of rodent-borne diseases in these populations. In turn, densely populated rodent communities near human settlements increase the risk of human-rodent contact, and subsequent transmission of rodent-borne disease agents to humans, creating favorable conditions for the emergence and spread of zoonoses [8]. Therefore, the risk of SEOV is indicative of inequality within society [9] as rat infestations in urban areas tend to increase with city growth and rising urban poverty [10]. Tropical and subtropical cities in America, Asia and Africa are particularly exposed to rodent-associated problems due to sometimes limited health and sanitary infrastructure combined with rapid population growth [11]. In Europe, SEOV detection (and transmission to humans) has been documented in pet rats [12] as well as in wild rat populations [13–15]. The strains detected in pet and wild rats are different [11] which raises questions about the origins of wild rats’ strains.

The presence of SEOV in France has been detected in both humans and rats previously [16,17]. The first SEOV-HFRS case detected in Europe, confirmed by molecular evidence, was found in a pregnant woman from Replonges [17], a locality close to Lyon, a densely populated city with over 500,000 inhabitants in France. Since then, several studies have demonstrated SEOV circulation in Lyon city area. Molecular evidence indicated the presence of SEOV in brown rats that were sampled in 2003 from colonies bred specifically for the study of rodenticides in Lyon [14]. The presence of SEOV was also confirmed by RT-PCR in 18/128 (14%) wild brown rats sampled between 2010–2012 in Lyon [18]. Complete genome sequences were obtained, which allowed to classify SEOV variants from Lyon within the lineage 7 [18].

The persistence and emergence or re-emergence of zoonotic pathogens in urban environments is a major concern for public and veterinary health. Cities and their peripheries are transportation hubs and are undergoing profound and accelerated socio-environmental changes [19]. This may lead to the profusion of rodents such as rats and mice in cities, and consequently, increased exposure of humans to rodent-borne zoonotic agents such as SEOV.

As only symptomatic treatment is available for hantavirus-associated diseases and considering their potential severity, preventing infections remains essential. This mainly consists in avoiding contact with rodents, their secretions and excretions or in implementing measures for controlling rodent populations. A better understanding of the ecological, epidemiological and evolutionary processes involved in the transmission of SEOV is required to mitigate the infection risk and to optimize prevention strategies. Furthermore, SEOV genomic sequences are critical for epidemiological surveillance by allowing the tracking of the spread and evolution of the virus.

We conducted in-depth investigation of SEOV circulation and genetic evolution in Lyon city. We focused on an urban park that is close to the place where SEOV was detected (positive rats detected between 2010–2012) and successfully sequenced from rats trapped in 2011 [18]. Due to its high popularity, the park is a hotspot for human/animal interactions, which makes the risk of SEOV transmission likely. We expected to find evidence of SEOV circulation in rats from this park due to the presence of the virus detected in the past in its vicinity [18], to the large population of rats in Lyon city and to the possibility of SEOV transmission between rats exploring the environment or looking for new territories. To test this hypothesis, we characterized SEOV seroprevalence and we analyzed the population genetics of *R*. *norvegicus*.

If the circulation of SEOV was confirmed, we were also interested in determining whether it is the same strain identified ten years after its initial detection or if it is a different strain introduced, possibly due to the widespread geographical dissemination of SEOV through fluvial transport or via potentially infected released pet animals. To unravel these hypotheses, we aimed to sequence complete S, M and L segments of SEOV in brown rats (*Rattus norvegicus*) surveyed in this park. We compared the SEOV genomes with the genetic data collected in the same area ten years ago to analyze the evolutionary history of SEOV strains in this urban park [18]. As such, this study provides a comprehensive serological and molecular characterization of SEOV in *Rattus norvegicus* from Lyon. It gives essential information to design surveillance strategies regarding SEOV risk in a popular urban park within a large city.

## Materials and methods

### Ethics statement

Rat capture and handling have been conducted according to the French and European regulations on care and protection of laboratory animals (French Law 2001–486 issued on June 6, 2001 and Directive 2010/63/EU issued on September 22, 2010). The Centre de Biologie pour la Gestion des Populations (CBGP) laboratory has approval (E-34-169-001) from the Departmental Direction of Population Protection (DDPP, Hérault, France), for the sampling of rodents and the storage and use of their tissues.

### Sampling

**Fig 1:**
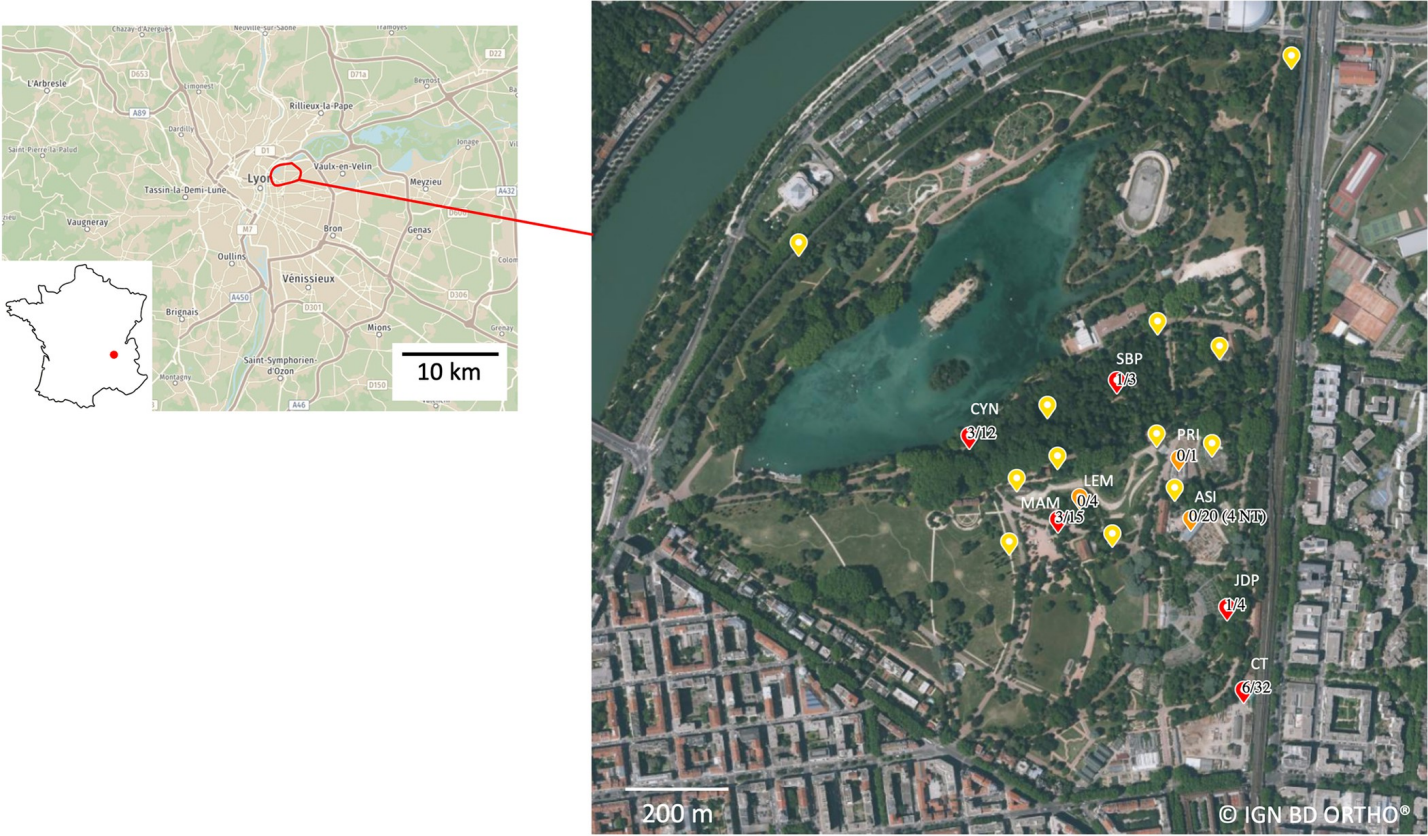
Map of the urban park of "la Tête d’Or” in Lyon, France. Yellow dots represent trap lines where no rats were caught. Orange dots represent trap lines where rats were trapped but none was seropositive for SEOV. Red dots represent trap lines where rats were trapped and some were SEOV seropositive. The codes of successful trap lines are indicated in white, and numbers indicate the proportion of SEOV seropositive rats. Copyright obtained from IGN.

Rats were collected during autumn 2020, spring and autumn 2021 and spring 2022 in the urban park of “la Tête d’Or” in Lyon city (Fig 1). The capture of animals was carried out in various types of habitats (wooded areas, buildings, zoological park, hay storage yards, riverside vegetation, restaurants, kids’ playgrounds, botanical garden and landfills), using live traps baited with sunflower seeds, carrots and sardine. Traps were set in places where animals had been previously detected (Fig 1), and they were removed after 10 or 11 nights. Each trap was geolocated. The traps were checked daily early in the morning. The rats were euthanized at the place of capture, by cervical dislocation and after sedation using isoflurane and the ’open drop’ method. Animal dissections and measurements were performed according to the protocols described in [20]. Sexual maturity was determined using individual weight and sexual characters (testes length and position, seminal vesicule development for males; uterus size, placental scars, presence of embryos and lactation for females). If at least one of these traits was developed, the rat was classified as mature. If none of these traits were developed, the individual was considered immature if its weight fell below 116g [21]. Capture data were registered in the small mammal database (BPM, http://bpm-cbgp.science), and the associated biological samples (such as organs, blood and parasites) were included in the CBGP reference collection of small mammals [22].

### Rat population genetics

DNA was extracted from a piece of kidney stored in 96% ethanol using BioBasic kit as recommended by the supplier. Rats were genotyped with 16 microsatellite loci (D2Rat97, D3Rat159, D4Rat59, D8Rat162, D10Rat105, D11Rat11, D12Rat49, D13Rat21, D14Rat110, D15Rat64, D18Rat11, D19Rat62, D20Mit4, D1VKC1A, D1VKC1C and D5Rat43) following the procedures described in [23]. Brown rats sampled in 2012 in Lyon city center and Givors, a city 30 km distant from Lyon, were added to the analyses. They had been trapped using live traps baited with peanut butter, oat flakes and sardine oil, checked daily. The captured rodents were killed by exposure to a rising concentration of carbon dioxide.

Indices of genetic diversity and genetic differentiation were estimated using the package *hierfstat* in R v4.1.3. The population genetic structure was also analysed through a discriminant principal component analysis (DAPC) [24] using the package *adegenet*.

### Indirect immunofluorescence assay

An indirect immunofluorescence assay (IFA) was used to detect antibodies to orthohantavirus in 87 rat blood samples as described previously [25,26]. The strain Orthohantavirus dobravaense was used in the IFA. Following the detection of IFA-positive samples, fourteen lung samples were directed to molecular detection.

### Library preparation and sequencing

Directly after the detection of antibodies to orthohantavirus, the lung samples of the seropositive rats were homogenized, and RNA was extracted using Trizol. No RT-PCR was performed. Ribosomal RNA was removed using NEBNext rRNA Depletion Kit (Human/Mouse/Rat), and the sequencing libraries were prepared using NEBNext Ultra II RNA library prep kit according to the manufacturer’s instructions. The samples were sequenced using NovaSeq 6000 System (Illumina). The virus sequences were detected and annotated using Lazypipe pipeline [27] using fastp for trimming adapters and low-quality bases, MEGAHIT v.1.2.8 for de novo assembly and MetaGeneAnnotator for the detection of gene-like regions. These were translated to amino acids with BioPerl and queried against the UniProtKB database using SANSparallel. This was followed by re-assembly against the de-novo assembled consensus sequences using BWA-MEM algorithm implemented in HaVoC pipeline [28].

### Phylogenetic analyses

Phylogenetic analyses were performed on three datasets composed of complete (or nearly-complete) coding regions of S, M and L segment sequences of SEOV recovered in this study and from sequences from different geographical areas available in GenBank. Sequences of Orthohantavirus hantanense, Orthohantavirus dobravaense and Orthohantavirus thailandense (Anjozorobe strain) were used as outgroups in all analyses. Gaps in the shorter sequences were treated as unknown characters (missing data). The likelihood at these sites is summed over all the possible states (i.e., nucleotides) that could be observed at these particular positions. Multiple sequence alignments were generated with the *Clustal* Omega alignment program implemented in *SeaView* v4.6.1. Phylogenetic analyses were then performed using the Maximum Likelihood method implemented in *PhyML* 3.0 (available at http://www.atgc-montpellier.fr/phyml/) with a statistical approximate likelihood ratio test (aLRT) for branch support. According to Guindon et al. 2010 [29], we considered that clade with SH-like aLRT supports higher than 0.8 were supported. The optimal substitution models were identified as the GTR+R model for all datasets using the “Automatic model selection by SMS” option implemented in *PhyML*. Phylogenetic trees were edited with *FigTree* v1.4.3.

Sequence identities were calculated with Sequence Identities and Similarities (SIAS) program available at (http://imed.med.ucm.es/Tools/sias.html).

### Sequence variation pattern analysis

The *Vespa* software [30] was used to detect sequence variations (amino-acids) between the SEOV strains obtained in this study relative to the sequence previously characterized from a rat trapped in Lyon city (SEOV_LYON/Rn/FRA/2013/LYO852).

## Results

### Genetic analyses of captured brown rats

**Fig 2:**
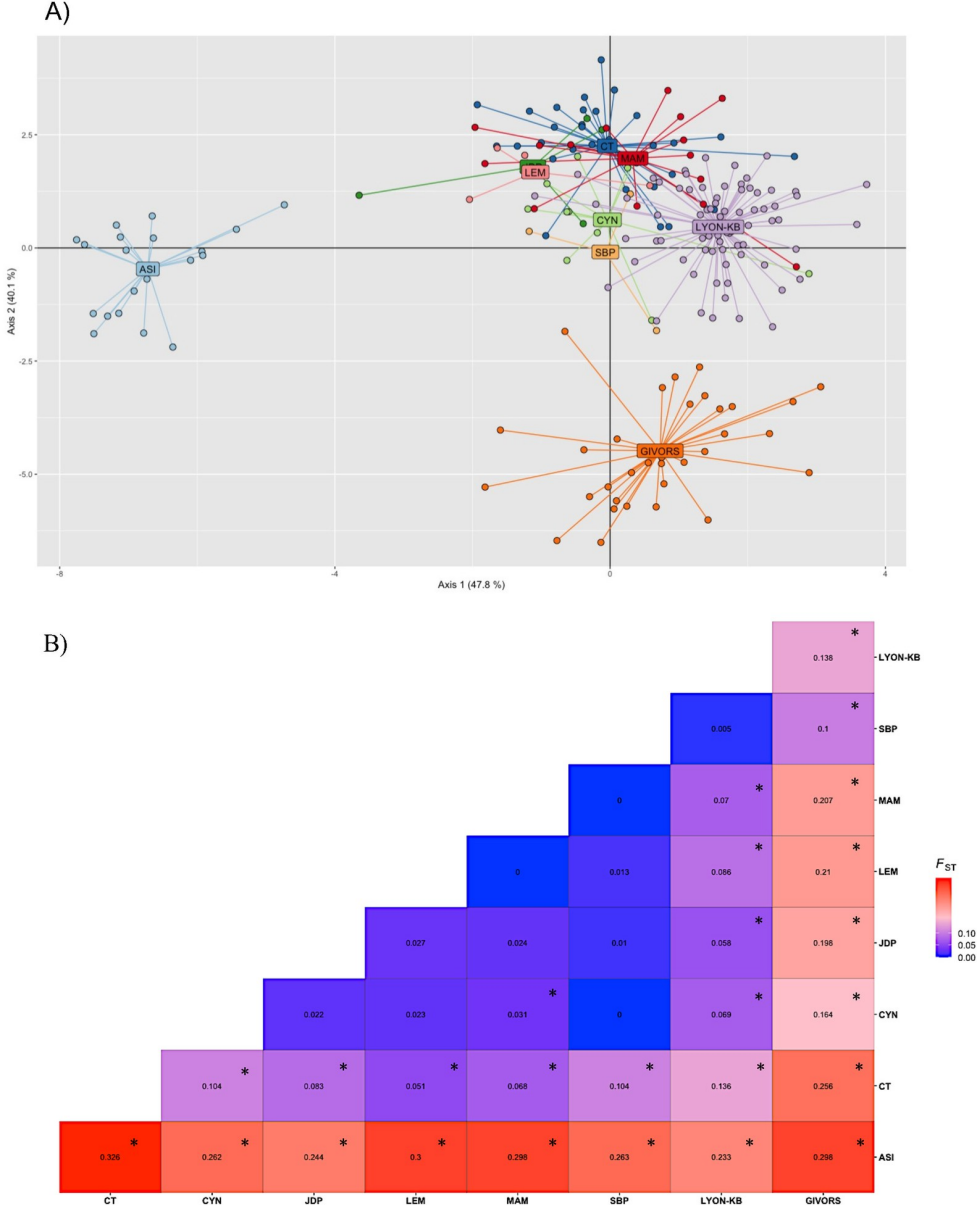
Genetic structuring of *R*. *norvegicus*. Assessment of the genetic structuring of *R*. *norvegicus* collected within the urban park “la Tête d’Or” in Lyon between 2020 and 2022 (trapping lines: ASI-light blue, LEM-pink, MAM-red, CYN-green, SBP-light orange, CT-dark blue, JDP-dark green), in Lyon city center (Lyon-KB-purple) and in Givors (orange; a city that is 30 km distant from Lyon), using a) DAPC and b) estimates of pairwise genetic differentiation (*F*_*ST*_). * corresponds to significant *F*_*ST*_ estimates (Fisher’s method)".

We successfully captured and genotyped 87 *R*. *norvegicus* from “la Tête d’Or”, and 107 from Lyon center and Givors, at the 16 microsatellites (S1 Table). Genetic diversity indices are reported in (Fig 2).

Pairwise estimates of genetic differentiation (*F*_ST_) ranged from 0.00 (between MAM and SBP trapping lines) to 0.13 (between Lyon center and CT line), but they reached higher values when comparing Givors to other sampling sites (0.10 to 0.29) and more surprisingly between ASI (a trapping line within the park) and all other sampling sites (0.24 to 0.32). The DAPC confirmed the high structuring between Lyon and Givors, and the low structuring within Lyon city, between trapping lines located within (except ASI) and outside the urban park. It also showed the strong genetic differentiation between the ASI trapping line and all other sampling sites from Lyon (Fig 2).

### Detection of orthohantavirus antibodies in brown rats

The circulation of SEOV was confirmed in the French rats from the urban park in Lyon as 15 out of 87 rats were seropositive for antibodies to orthohantavirus (Table 1). The overall seroprevalence over the period 2020–2022 was 17.2%. Only one rat among the 15 seropositive ones was a juvenile (and therefore a potential carrier of maternal antibodies), so that SEOV serology was significantly influenced by rat maturity (Fisher’s exact test, *p* = 0.03). The difference in SEOV seroprevalence between males and females was not significant (X^2^ = 1.75e-31, *p* = 1).

**Table 1:** Rat captures for each trap line and trapping period. Numbers between brackets correspond to the number of seropositive rats.

Trap line	Autumn 2020	Spring 2021	Autumn 2021	Spring 2022	Total
**ASI**	0	0	4 (0)	12 (0)	16 (0)
**CT**	0	2 (1)	11 (2)	19 (4)	32 (7)
**CYN**	0	7 (2)	2 (0)	3 (1)	12 (3)
**JDP**	1 (0)	1 (1)	1 (0)	1 (0)	4 (1)
**LEM**	4 (0)	0	0	0	4 (0)
**MAM**	7 (0)	4 (3)	4 (0)	0	15 (3)
**PRI**	0	0	1 (0)	0	1 (0)
**SBP**	0	1 (1)	2 (0)	0	3 (1)

### Phylogenetic analysis of SEOV strains

Only the SEOV seropositive rats were sequenced on the Illumina NovaSeq 6000 Sequencing platform, which resulted in 3.1–6.0 Gb per sample (S2 Table). We recovered seven complete or nearly complete genomes of SEOV from those individuals, with 6453 nt for the L-segment, 3399 nt for the M-segment (all individuals), and 1287 nt for the S-segment (seven individuals). We could not retrieve any SEOV genomes from the eight other ones. GenBank numbers were obtained for all the corresponding SEOV segments recovered in this study (S3 Table).

**Fig 3:**
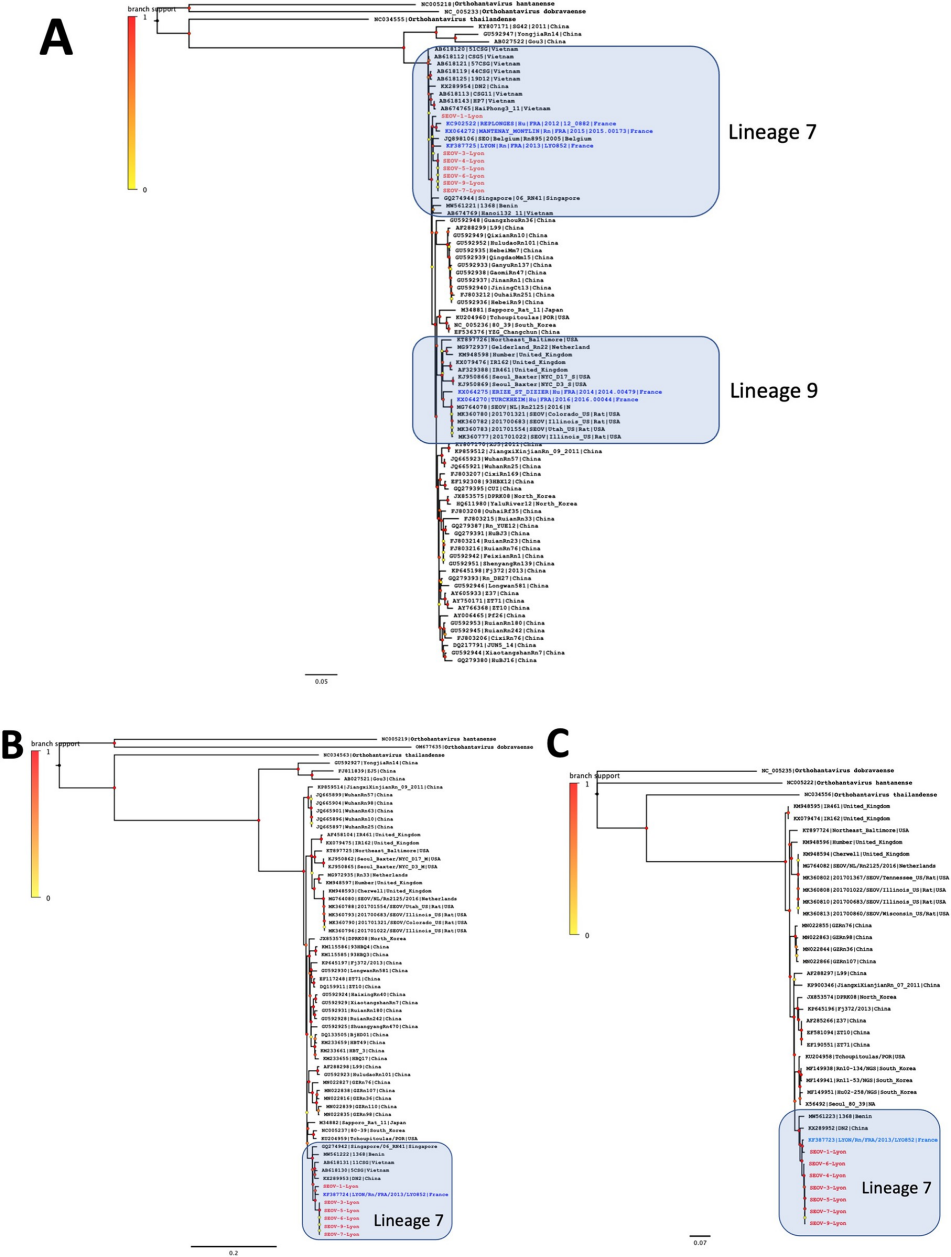
Phylogenetic analysis of SEOV. The phylogenetic analysis of SEOV gene segments recovered from brown rats (*Rattus norvegicus*) trapped in Lyon city, France (in red) and reference sequences (other sequences from France are represented in blue). Phylogenetic trees were generated with PhyML 3.0 by the maximum-likelihood method on the complete coding region of the small (A), medium (B) and large (C) segments with a GTR + R nucleotide substitution model. Branch support as determined by an aLRT test is represented by a color dot at each node. Scale bars indicate n of substitutions per nucleotide. Only lineages 7 and 9 were highlighted in the phylogenetic trees.

The phylogenetic analysis showed that SEOV harbored by wild rats (*Rattus norvegicus*) in the urban park of “la Tête d’Or” in Lyon (sequences hereafter named SEOV-X-Lyon) were closely related to those of rat-derived viral genomes identified previously in Lyon city (Fig 3).

All the SEOV strains from this study grouped within the previously defined lineage 7 [18] which includes strains from Europe (France, Belgium), South-East Asia (Singapore and Vietnam) and West Africa (Benin). The phylogenies of S, M, and L segments of the seven SEOV strains obtained in this study showed high phylogenetic proximity with strains previously collected in Lyon city (SEOV_LYON/Rn/FRA/2013/LYO852) or from its vicinity (REPLONGES|Hu|FRA|2012|12_0882). Particularly, the strain SEOV-1 (marked in red color) was closer to the sequence SEOV_LYON/Rn/FRA/2013/LYO852 than to the other strains identified in this study, whatever the phylogeny considered (S, M, or L segments, Fig 3). Interestingly, the viral genomes derived from humans and rats obtained in France clustered with lineages 7 and 9 (marked in blue), what indicates the circulation of at least two lineages of SEOV in France so far.

**Fig 4:**
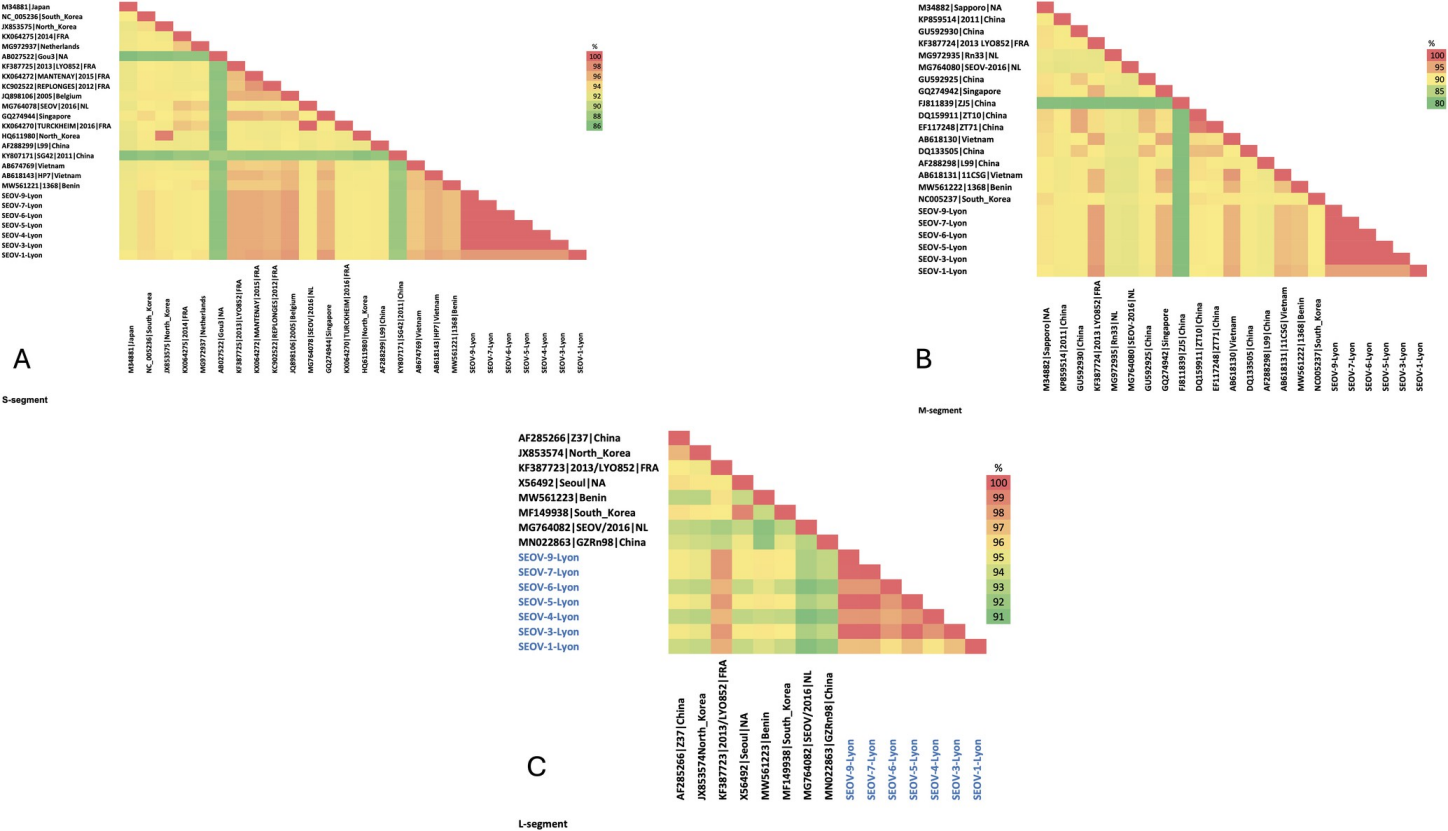
Pairwise protein sequence identification. The pairwise protein sequence identities calculated with the SIAS software for (A) the nucleocapsid protein, (B) the glycoprotein precursors and (C) the RdRp. Analyses were performed considering mainly sequences belonging to lineage 7 and two SEOV-human reference sequences from France belonging to lineage 9.

The SEOV strains from Lyon showed very high similarity at the amino-acid level (Fig 4). The identities were 100% for the nucleocapsid protein, between 99.4% and 100% for glycoprotein precursors and between 97.2% and 100% for the RdRp. The SEOV strains detected in this study also showed high genetic similarity with the strain previously sequenced from a rat in Lyon (LYON/Rn/FRA/2013/LYO852; identity of 100% for the nucleocapsid protein, 99.4% to 99.5% for the glycoprotein precursors and 98.3% to 99.8% for the RdRp).

### Amino acid sequences variation analysis

We identified amino acid variations between the SEOV strains detected in this study compared to the strain identified previously (SEOV_LYON/Rn/FRA/2013/LYO852). We looked for evidence of amino acid substitutions between SEOV strains in Lyon between 2011 and 2021 (S1 Appendix). Only mutations that were detected in all seven newly sequenced strains were considered suggestive of fixed mutations in the currently circulating SEOV strains. We did not detect any pattern of mutation for the nucleocapsid protein (see S1 Appendix). For the glycoprotein precursor and RdRp, three and two amino acid exchange, respectively, were detected in all the newly sequenced strains compared to SEOV_LYON/Rn/FRA/2013/LYO852 strain: I953V (conservative mutation), L1051S (non-conservative mutation), F1113L (conservative mutation) in Glycoprotein precursor, and T330A (conservative mutation), I1775V (conservative mutation) in RdRp. According to studies based on other closely related hantaviruses, amino acid exchanges are located in domain I, III and in the C-terminal transmembrane anchor of the Gc [31] and in the polymerase core and in the presumed cap binding domain (CBD) of the RdRp [32]. The majority of these substitutions are conservative, indicating that they likely do not significantly affect the stability or function of the proteins [33].

## Discussion

SEOV as a human pathogen, has been circulating between urban rats and that represents a significant threat to public health, particularly in urban areas where the densities of rodent populations are high. As cities continue to grow and expand, the risk of SEOV transmission is likely to increase. It is therefore essential to develop effective surveillance and control strategies, using an ecohealth approach, to prevent and manage SEOV outbreaks in urban areas. Such ecohealth approach should include the inter- and transdisciplinary study and surveillance of the ecological, environmental, and health factors that contribute to the transmission of the virus. As such, a comprehensive assessment of the ecology of rats and SEOV is required. A deepened knowledge of the ecosystem (small mammal biodiversity, microbial diversity including rat-borne zoonotic agents such as *Leptospira* sp., hepatitis E virus, *Streptobacillus moniliformi*s, and environmental conditions and changes) is critical as it has strong impacts on SEOV survival and transmission. Lastly, understanding how humans interact with the natural environment and potential sources of SEOV exposure is necessary to design preventive measure and information campaigns. Overall, this ecohealth approach should facilitate the mitigation of SEOV risk by reducing the circulation of the virus, decreasing human exposure, and raising awareness among the public and healthcare professionals. It should also facilitate the design of rat control strategies aimed at keeping their abundance below a specific threshold, thereby reducing SEOV transmission and mitigating other issues associated with the proliferation of these rodents.

SEOV is considered as an underdiagnosed virus due to the limited diagnostic assays and, presumably, unawareness among physicians. Indeed, although four orthohantaviruses (Puumala [PUUV], SEOV, Tula, and Nova viruses) have been identified in France, PUUV is the most strongly associated with human infections. Infections with SEOV are likely to be missed in France, either because hantavirus infection is rarely suspected outside the PUUV endemic area (North-Eastern France), or because most of the public hospital or private clinical laboratories in France use commercial kits for hantavirus serologic diagnosis directed against PUUV (POC Puumala IgM rapid test [Reagena, Toivala, Finland]). Only few hospitals use IgM and IgG ELISA kits with mixtures of recombinant antigens, including those from SEOV or Hantaan virus strains [16]. Continuous surveillance is needed to trace the virus spread, circulation and evolution in wild and domestic rats to understand the risk and to reduce that risk of SEOV outbreak in humans. In this context, we studied the circulation and evolution of SEOV among wild brown rats in a popular urban park in Lyon city (France), where SEOV circulation was detected ten years ago [18]. This park is a multifacility area with a lake, a zoo, a botanical garden, restaurants, horses’ paddocks, garbage dump and carousels. Opportunities for SEOV transmission from rats to domestic animals or humans are thus numerous. Since the detection of SEOV in wild urban rats in Lyon [18], there had not been any eco-epidemiological or evolutionary survey of SEOV in this area. It was therefore critical to pursue the eco-evolutionary surveillance of this virus, to provide an efficient early warning system and prepare risk-based interventions that target people and areas at highest risk of exposure to infected rats.

In total, 87 brown rats were caught from different sites within the urban park between 2020 and 2022. The detected overall seroprevalence (17.2%) was quite high and comparable to what has been observed in other cities [9]. Nevertheless, reports on SEOV seroprevalence in Lyon city are limited; therefore, it should be followed. All seropositive rats except one were sexually mature, and they were trapped in several sampling sites within the park. This confirms the continuous and high level of SEOV circulation in rats in this urban park since at least 2010–2012 [18], although no human case has been detected yet.

We have added to the analysis brown rats sampled in 2012 in Lyon city center and Givors, a city 30 km distant from Lyon. Including these samples enabled to detect potential barriers to gene flow between rats from the park and those from the city, which could indicate a potential limitation in SEOV transmission between subpopulations. Besides, Givors samples were added to give insights into the expected levels of genetic differentiation between geographically distant rat populations. The genetic analyses of rats have revealed a main cluster consisting of rats from both the park and the city center of Lyon. This suggests a large rat population and possibility of gene flow between the park and the rest of the city. The likelihood of released pet rats establishing colonies in the park is extremely low. The rats that were trapped did not display ’pet-like’ morphotypes, such as particular color patterns. The park’s high population abundance of wild rats and the presence of large mammals, including carnivores in the zoo, should prevent the settlement of ’pet rat’ colonies. As a result, SEOV may potentially circulate and evolve throughout Lyon, so that SEOV surveillance should be conducted at the scale of the city. Ecological surveys should also be conducted to evaluate rat movements within the park, and between the park and adjacent areas of Lyon city. This will enhance our understanding of the geographical scale and seasonal timing at which SEOV transmission takes place.

Interestingly, rats trapped in the ‘Asian forests’ building of the zoological park (ASI), an area opened in 2021 to home a large diversity of mammals and birds, showed high levels of genetic differentiation compared to the rats trapped in other parts of the park. They were all seronegative. This could suggest a recent introduction, or a strong founder effect associated with the creation of this area. It should be important in the future to pay particular attention to this site, to survey the evolution of rat populations and SEOV prevalence in rats and captive monkeys (*Nomascus leucogenys*) of the ‘Asian forests’ building.

We recovered seven complete and semi-complete coding regions of SEOV from these wild rats, which greatly increase the genetic data available on SEOV from France, especially for the M and L segments. The recovered genomes were significantly larger in contrast to the previous studies [16–18]. Genetic analyses showed high identity levels between all these sequences. Indeed, only a few amino acid substitutions were identified between 2010 and 2021, on the glycoprotein precursor and the RdRp. This suggests strong purifying selection as already described [34]. Note that the lack of complete genomes available prior to this study requires considering these results with caution.

Phylogenetic analyses revealed that all SEOV strains retrieved in this study clustered together with the recent SEOV strain from Lyon (SEOV_LYON/Rn/FRA/2013/LYO852) with SEOV-1 being slightly more distant from the others. This sheds light on the origin of SEOV strains circulating in France and in Lyon city. The strains described in this study clustered together with strains from Asia (Vietnam, Singapore, and China) and the recently sequenced one from Africa (Benin) within the SEOV lineage 7 [23]. South-East Asia was suggested to be the origin of lineages 7 and 9 that are circulating in Europe and causing infections in humans [35]. Both lineages were identified in France [16], with a human case associated with lineage 7 near Lyon [17], suggesting multiple SEOV introduction events in France. Identifying the origin of the SEOV strains circulating in Lyon remains a primary question. This knowledge is fundamental to design further management strategies that will enable to limit rat populations by better controlling introductions, potentially associated with commercial trade (in particular with Southeast Asia, [4,15,36,37].

Taken together, these findings suggest that the same strain of SEOV has been circulating continuously in Lyon since at least 2011 at a significant seroprevalence, with only a few new fixed mutations detected in 2020–2022. Our results underscore the importance of regularly monitoring SEOV strains present in rats in urban areas like Lyon. Urban brown rats could be considered sentinels for the active surveillance of SEOV and other targeted pathogens such as viruses, bacteria, protozoa, and helminths in city. Enhanced surveillance of SEOV transmission among rats can assist in better preventing and identifying potential outbreaks in humans, which is critical in mitigating the associated zoonotic risk. In addition, it is now critical to evaluate the risk of SEOV for public and veterinary health, by assessing human and captive animals’ exposure to SEOV using serological surveys, as recently performed for PUUV risk for example [38].

## Supporting information

S1 Table Rattus norvegicus individual information. (XLSX)

S2 Table Results provided by the NovaSeq platform. (DOCX)

S3 Table The Code sequence and GenBank number together with the Code manuscript. (DOCX)

S1 Appendix Frequencies of amino-acids of the query set and background set sequences. The query set (SEOV strains derived from the present study) and the background sequences (SEOV_LYON/Rn/FRA/2013/LYO852 sequences derived from the database GenBank). a) Signature of codons detected on the SEOV M-segment. b) Signature of codons detected on the SEOV L-segment. The first upper line shows the query signature amino-acids and the two lines below show the frequency of those amino-acids among the query set and the background set, respectively. The fourth line illustrates the common amino-acids detected among the background set. The following two lines beneath show the frequency of those amino-acids among the query set and the background set sequences, respectively. The last line refers to the alignment position among those sequences. The figures under each table show the amino-acid variations between the query set (upper part) and the background set (lower part).
(PPTX)
