# Supplementary material for: Evolution and genetic characterization of Seoul virus in wild rats Rattus norvegicus from an urban park in Lyon, France 2020–2022
Source: PLoS Negl Trop Dis. 2024 May 13;18(5):e0012142. doi: 10.1371/journal.pntd.0012142 (PMC11149884; doi:10.1371/journal.pntd.0012142)
Supplement: S3 Table — (DOCX) [file pntd.0012142.s003.docx]

**Evolution and Genetic Characterization of Seoul Virus in Wild Rats *Rattus Norvegicus* from an urban park in Lyon, France 2020-2022**

﻿Short Title: Seoul virus in wild rats from an urban park

Authors : Alburkat Hussein, Smura Teemu, Bouilloud Marie, Pradel Julien, Anfray Gwendoline, Berthier Karine, Dutra Lara, Loiseau Anne, Niamsap Thanakorn, Olander, Viktor, Sepulveda Diana, Venkat Vinaya, Charbonnel Nathalie, Castel Guillaume, Sironen Tarja

**S3 Table :** **The Code sequence and GenBank number together with the Code manuscript.**

| Code manuscript | Code sequence | Segment | Genbank number | Direct link to the repository GenBank |
| --- | --- | --- | --- | --- |
| SEOV-1 | NCHA001216 | S | OQ865194 | <https://www.ncbi.nlm.nih.gov/nuccore/OQ865194> |
|  |  | M | OQ865200 | <https://www.ncbi.nlm.nih.gov/nuccore/OQ865200> |
|  |  | L | OQ865208 | <https://www.ncbi.nlm.nih.gov/nuccore/OQ865208> |
| SEOV-3 | NCHA001225 | S | OQ865193 | <https://www.ncbi.nlm.nih.gov/nuccore/OQ865193> |
|  |  | M | OQ865199 | <https://www.ncbi.nlm.nih.gov/nuccore/OQ865199> |
|  |  | L | OQ865207 | <https://www.ncbi.nlm.nih.gov/nuccore/OQ865207> |
| SEOV-4 | NCHA001251 | S | OQ865192 | <https://www.ncbi.nlm.nih.gov/nuccore/OQ865192> |
|  |  | M | OQ865201 | <https://www.ncbi.nlm.nih.gov/nuccore/OQ865201> |
|  |  | L | OQ865206 | <https://www.ncbi.nlm.nih.gov/nuccore/OQ865206> |
| SEOV-5 | NCHA001256 | S | OQ865191 | <https://www.ncbi.nlm.nih.gov/nuccore/OQ865191> |
|  |  | M | OQ865198 | <https://www.ncbi.nlm.nih.gov/nuccore/OQ865198> |
|  |  | L | OQ865205 | <https://www.ncbi.nlm.nih.gov/nuccore/OQ865205> |
| SEOV-6 | NCHA001262 | S | OQ865190 | <https://www.ncbi.nlm.nih.gov/nuccore/OQ865190> |
|  |  | M | OQ865197 | <https://www.ncbi.nlm.nih.gov/nuccore/OQ865197> |
|  |  | L | OQ865204 | <https://www.ncbi.nlm.nih.gov/nuccore/OQ865204> |
| SEOV-7 | NCHA001324 | S | OQ865189 | <https://www.ncbi.nlm.nih.gov/nuccore/OQ865189> |
|  |  | M | OQ865196 | <https://www.ncbi.nlm.nih.gov/nuccore/2562832785> |
|  |  | L | OQ865203 | <https://www.ncbi.nlm.nih.gov/nuccore/OQ865203> |
| SEOV-9 | NCHA002108 | S | OQ865188 | <https://www.ncbi.nlm.nih.gov/nuccore/OQ865188> |
|  |  | M | OQ865195 | <https://www.ncbi.nlm.nih.gov/nuccore/OQ865195> |
|  |  | L | OQ865202 | <https://www.ncbi.nlm.nih.gov/nuccore/OQ865202> |
