# Supplementary material for: Evolution and genetic characterization of Seoul virus in wild rats Rattus norvegicus from an urban park in Lyon, France 2020–2022
Source: PLoS Negl Trop Dis. 2024 May 13;18(5):e0012142. doi: 10.1371/journal.pntd.0012142 (PMC11149884; doi:10.1371/journal.pntd.0012142)
Supplement: S1 Appendix — The query set (SEOV strains derived from the present study) and the background sequences (SEOV_LYON/Rn/FRA/2013/LYO852 sequences derived from the database GenBank). a) Signature of codons detected on the SEOV M-segment. b) Signature of codons detected on the SEOV L-segment. The first upper line shows the query signature amino-acids and the two lines below show the frequency of those amino-acids among the query set and the background set, respectively. The fourth line illustrates the common amino-acids detected among the background set. The following two lines beneath show the frequency of those amino-acids among the query set and the background set sequences, respectively. The last line refers to the alignment position among those sequences. The figures under each table show the amino-acid variations between the query set (upper part) and the background set (lower part). (PPTX) [file pntd.0012142.s004.pptx]

## Slide 1
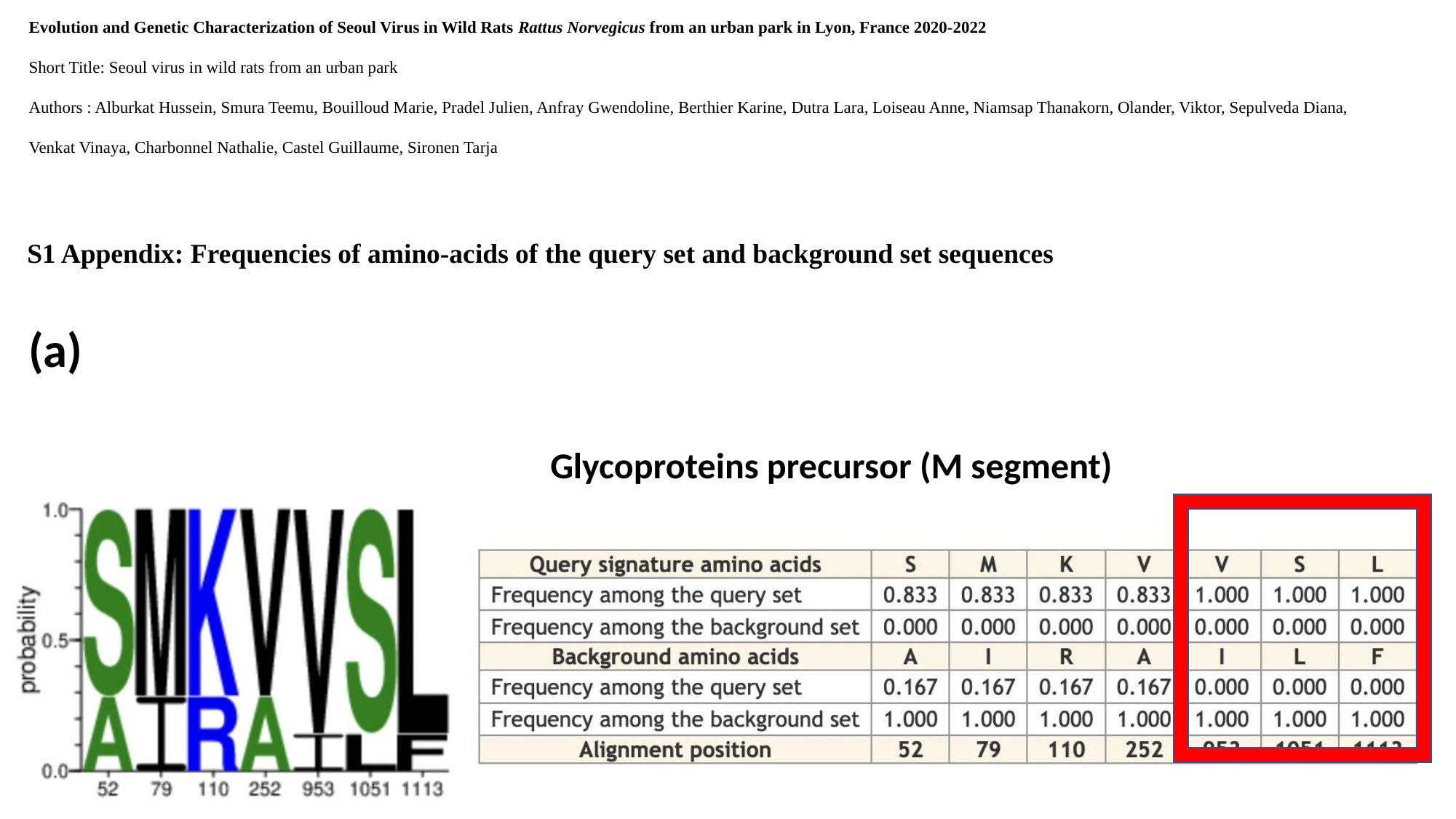

Evolution and Genetic Characterization of Seoul Virus in Wild Rats Rattus Norvegicus from an urban park in Lyon, France 2020-2022
Short Title: Seoul virus in wild rats from an urban park
Authors : Alburkat Hussein, Smura Teemu, Bouilloud Marie, Pradel Julien, Anfray Gwendoline, Berthier Karine, Dutra Lara, Loiseau Anne, Niamsap Thanakorn, Olander, Viktor, Sepulveda Diana, Venkat Vinaya, Charbonnel Nathalie, Castel Guillaume, Sironen Tarja
S1 Appendix: Frequencies of amino-acids of the query set and background set sequences
(a)
Glycoproteins precursor (M segment)

## Slide 2
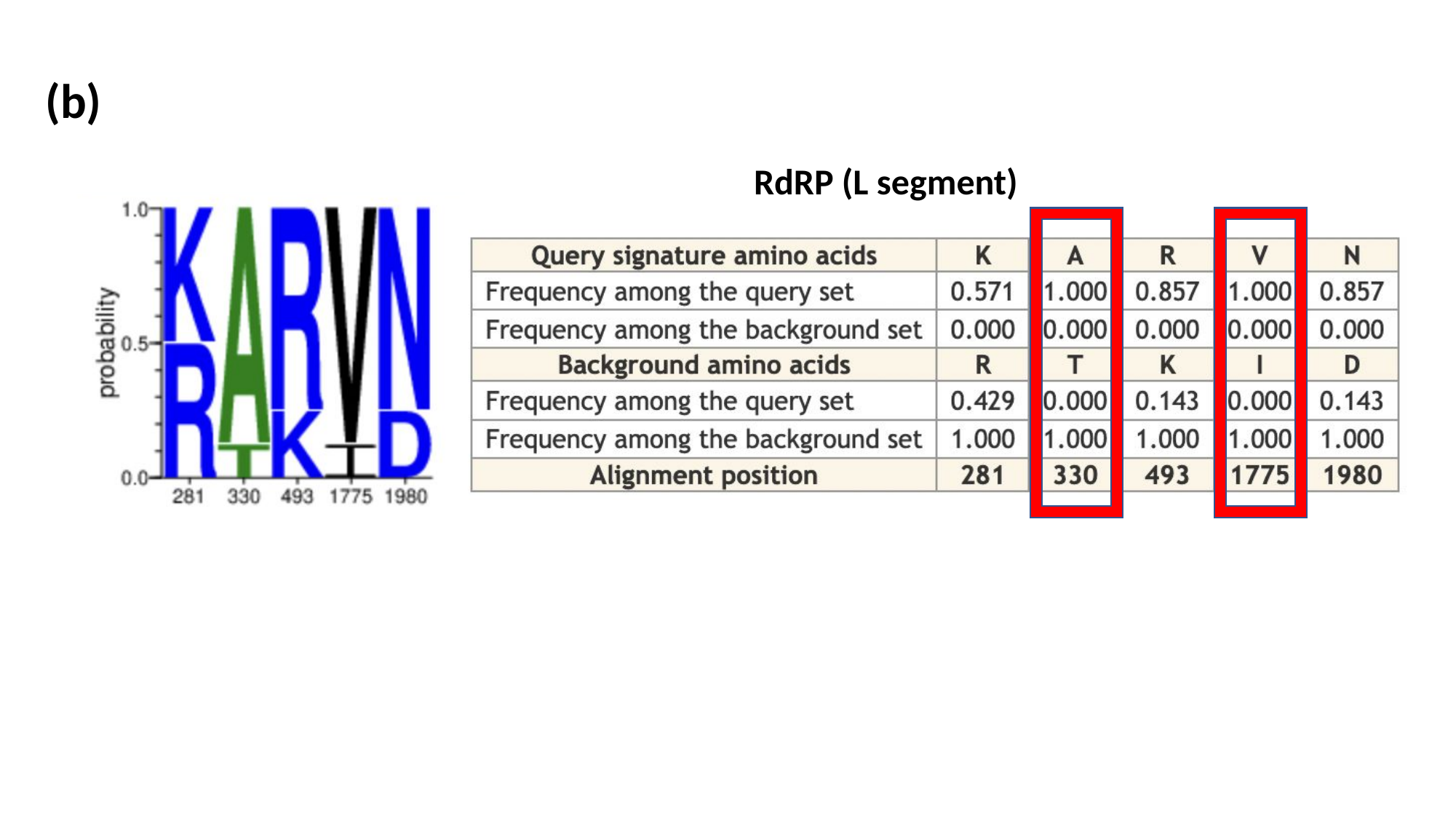

(b)
RdRP (L segment)
